# Low Serum Uric Acid as an Independent Predictor of Mortality and Poor Prognosis: A Retrospective Cohort Study

**DOI:** 10.3390/jcm14196855

**Published:** 2025-09-27

**Authors:** Seher İrem Şahin, Ece Çiftçi Öztürk, Hüseyin Öztürk, Büşra Çetintulum Aydın, Fatma Pınar Ziyadanoğlu Cezairli, Emre Hoca, Hayriye Esra Ataoğlu

**Affiliations:** 1 Department of Internal Medicine, Istanbul Haseki Training and Research Hospital, Istanbul 34270, Turkey; eciftci3506@gmail.com (E.Ç.Ö.); busracetintulum@hotmail.com (B.Ç.A.); pinarziyadanoglu@hotmail.com (F.P.Z.C.); emrehoca89@gmail.com (E.H.); eataoglu@gmail.com (H.E.A.); 2 Department of Internal Medicine, Basaksehir Cam and Sakura City Hospital, Istanbul 34480, Turkey; huseyinozturkdr@gmail.com

**Keywords:** chronic kidney disease, hypouricemia, ICU admission, mortality, prognosis, uric acid

## Abstract

**Background:** While hyperuricemia has been widely studied in cardiovascular and renal diseases, the prognostic impact of low serum uric acid (UA) remains unclear. Emerging evidence suggests hypouricemia may be linked to increased mortality and adverse outcomes. This study aimed to assess the relationship between low UA levels and poor outcomes, including mortality and intensive care unit (ICU) admission, in hospitalized patients. **Methods:** This retrospective cohort study included 1679 hospitalized patients (744 females, 935 males) from the Internal Medicine Clinic. Patients were categorized into normal and low UA groups based on sex-specific thresholds (male: <3.4 mg/dL, female: <2.4 mg/dL). The primary outcome was in-hospital mortality; secondary outcomes were ICU admission and discharge status. Logistic regression models adjusted for age, chronic kidney disease (CKD), hypertension (HT), and coronary artery disease (CAD). A Prognostic Uric Acid Score (PUAS) was developed using significant predictors and evaluated by Receiver Operating Characteristic (ROC) analysis. **Results:** Low UA levels were significantly associated with higher ICU admission and mortality (*p* = 0.012). Multivariate analysis identified age (OR: 1.032), low UA (OR: 2.285), and CKD (OR: 1.571) as predictors of poor prognosis. PUAS showed moderate performance (AUC: 0.664), with a cutoff score of 3.5 optimizing sensitivity and specificity. **Conclusions:** Low UA levels independently predict mortality and poor prognosis in hospitalized patients. These findings support routine UA monitoring and suggest hypouricemia may be a useful prognostic biomarker. Further studies are needed to understand clinical implications and guide UA-targeted interventions.

## 1. Introduction

Uric acid (UA) is the final metabolite of purine metabolism in humans, as the evolutionary loss of uricase activity has led to its accumulation [1]. UA has been identified as a key mediator in various pathological processes, including oxidative stress, inflammation, and endothelial dysfunction [2]. Hyperuricemia is commonly defined as a serum UA level ≥ 7.0 mg/dL in men and ≥6.0 mg/dL in women, while hypouricemia is typically classified as ≤2.5 mg/dL, although no universally accepted definition exists. Serum UA levels are regulated by a dynamic balance involving dietary purine intake, endogenous purine metabolism, renal reabsorption and excretion, and intestinal elimination [3].

The relationship between elevated UA levels and cardiovascular events was first highlighted in the Framingham Heart Study (1967), which demonstrated an increased risk of myocardial infarction in individuals with hyperuricemia over a 12-year follow-up period [4]. Similarly, the Rotterdam Study identified hyperuricemia as a risk factor for both fatal and nonfatal acute coronary syndrome in the general population [5].

While much of the existing literature has focused on the adverse effects of hyperuricemia, emerging evidence suggests that low UA levels may also be associated with poor clinical outcomes. A large cohort study from Japan reported that low UA levels (<4.6 mg/dL in men and <3.3 mg/dL in women) were linked to increased cardiovascular mortality [6]. Furthermore, multiple studies have demonstrated a significant association between hypouricemia and increased all-cause mortality, particularly in patients undergoing dialysis [7,8].

UA is a potent antioxidant, and its depletion has been implicated in endothelial dysfunction, increased oxidative stress, and heightened susceptibility to conditions such as hypertension, diabetes mellitus, and chronic kidney disease [9,10]. These findings raise important clinical questions regarding the role of low UA levels as a potential prognostic marker.

While hyperuricemia has been extensively studied in relation to cardiovascular and renal diseases, hypouricemia remains an underexplored entity with potential prognostic significance. Its impact on patient outcomes, particularly in hospitalized individuals, warrants further investigation. Given these associations, our study aims to evaluate the impact of low serum UA levels on patient prognosis, specifically assessing its relationship with mortality and adverse clinical outcomes.

## 2. Materials and Methods

### 2.1. Study Population

This retrospective cohort study analyzed electronic health records of patients aged ≥18 years who were hospitalized in the Internal Medicine Clinic in 2019. Patients were classified based on serum UA levels according to the hospital’s reference ranges. Those with normal or low UA levels were eligible for inclusion. For males, the normal serum UA range was 3.4–7.0 mg/dL, and for females, it was 2.4–5.7 mg/dL. Patients with serum UA levels < 3.4 mg/dL in males and <2.4 mg/dL in females were classified as having low UA levels.

**Figure 1:**
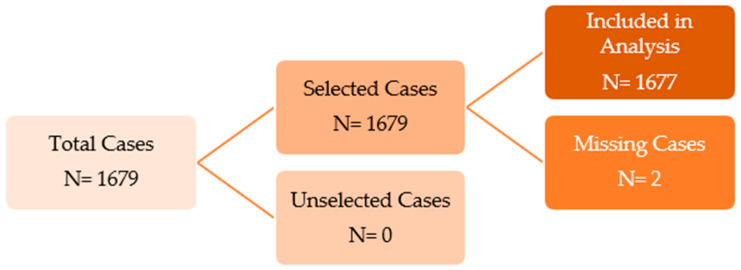
Case Processing Summary.

A total of 1679 patients (744 females and 935 males) met the inclusion criteria. A case processing summary is shown in the diagram below (Figure 1).

### 2.2. Exclusion Criteria

The exclusion criteria were as follows:

1. Use of medications affecting UA metabolism, including uric acid-lowering agents (allopurinol, febuxostat), diuretics, and aspirin (>325 mg/day).
2. Presence of end-stage renal disease (ESRD, CKD stage 4–5) or decompensated heart failure (NYHA class III–IV).
3. A confirmed diagnosis of malignancy (any type of cancer).

### 2.3. Laboratory Measurements and Data Collection

Patient data, including age, sex, chronic disease status, medication use, admission diagnosis, hospital stay duration, Intensive Care Unit (ICU) admission, and mortality status, were collected from the hospital’s electronic medical records.

Serum UA levels were measured using an enzymatic colorimetric method. Routine biochemical parameters, including urea, creatinine, fasting blood glucose, albumin, alanine aminotransferase (ALT), aspartate aminotransferase (AST), and C-reactive protein (CRP), were analyzed using an automated biochemical analyzer.

Chronic kidney disease (CKD) stages were classified according to KDIGO guidelines (stages 1–3). Serum uric acid (UA) levels were stratified across these stages to assess potential prognostic differences. Patients with advanced CKD (stages 4–5) were excluded due to the profound metabolic alterations associated with end-stage renal disease.

Information regarding the use of sodium-glucose cotransporter-2 inhibitors (SGLT2i) was not systematically available in our dataset and therefore not included in the analysis. Similarly, detailed information on episodes of acute kidney injury (KDIGO stages I–III) was not collected and thus could not be evaluated.

### 2.4. Ethical Considerations

This study adhered to the principles of the Declaration of Helsinki for human research ethics and received approval from the Istanbul Haseki Training and Research Hospital Ethics Committee (Approval No: 28-2023). Patient data were anonymized to ensure confidentiality.

### 2.5. Outcome Measures

The primary outcome was in-hospital mortality. Secondary outcomes included ICU admission and discharge status. Poor prognosis was defined as either ICU admission or mortality. The study aimed to assess the association between low serum UA levels and clinical outcomes.

### 2.6. Statistical Analysis

Descriptive statistics were used to summarize the demographic and clinical characteristics of the study population. Continuous variables were expressed as mean ± standard deviation (SD) or median (interquartile range, IQR) based on their distribution, assessed using the Shapiro–Wilk test. Categorical variables were presented as frequencies and percentages.

Comparisons between groups (normal vs. low UA levels) were performed using the chi-square test (χ^2^) for categorical variables and the independent samples *t*-test or Mann–Whitney U test for continuous variables.

To evaluate the association between low UA levels and poor prognosis (ICU admission and mortality), univariate and multivariate logistic regression analyses were conducted. The multivariate model included adjustments for age, CKD, HT, and CAD. The results were expressed as odds ratios (ORs) with 95% confidence intervals (CIs).

To develop the Prognostic Uric Acid Score (PUAS), a risk scoring system based on significant variables was constructed. The discriminative ability of PUAS was assessed using ROC curve analysis, and the area under the curve (AUC) was calculated. The optimal cutoff value was determined using the Youden Index.

A *p*-value < 0.05 was considered statistically significant. All statistical analyses were performed using SPSS (IBM SPSS Statistics, version 22).

## 3. Results

A total of 1679 patients were included in the study. However, two cases were missing and were excluded from the final analysis. In our study conducted on hospitalized patients, the need for intensive care unit admission and death were defined as poor prognosis following hospitalization.

Among the study participants, 1480 individuals had normal UA levels, and 93% of this group was discharged while 7% had poor prognosis. In comparison, 197 individuals had low UA levels, and 87.8% of this group was discharged, and 12.2% had poor prognosis. According to these results a statistically significant association was found between UA levels and both discharge status and poor prognosis (χ^2^ test, *p* < 0.05).

The table below presents the distribution of patients based on key clinical variables and their associated categories. It categorizes patients according to the presence or absence of conditions such as Chronic Kidney Disease (CKD), Hypertension (HT), and Coronary Artery Disease (CAD). Additionally, it includes patients classified by UA Levels, with distinct groups for normal and low levels. The table also provides the distribution of patients in the prognosis category, further divided into those who were discharged and those with a poor prognosis (Table 1).

Based on the data presented in Table 1, the prognosis of patients in terms of discharge status and poor outcomes, is significantly influenced by several clinical variables, including age, the presence of chronic kidney disease (CKD), and serum UA levels.

The median age of patients who were discharged was 57 years, while those with poor prognosis had a significantly higher median age of 65 years (*p*-value < 0.001). This finding suggests that advanced age is associated with worse clinical outcomes.

The presence of CKD was associated with a worse prognosis. Among patients without CKD, 93% were discharged, whereas only 88% of patients with CKD were discharged. Conversely, 12% of patients with CKD had poor prognosis, compared to only 7% of those without CKD. The difference was statistically significant (*p*-value = 0.015), indicating that CKD is a risk factor for worse outcomes.

When stratified by CKD stage, according to the KDIGO classification mean serum uric acid levels showed significant differences across groups (*p* < 0.001). Patients with normal renal function (G1, eGFR ≥ 90 mL/min/1.73 m^2^) had the lowest mean UA levels (4.08 mg/dL) [11]. UA levels increased in stages G2 (5.10 mg/dL), G3a (5.35 mg/dL), and G3b (5.29 mg/dL), but declined again in advanced CKD stages G4 and G5 (both 4.88 mg/dL). Post hoc Bonferroni analysis revealed that UA levels in G1 were significantly lower compared to all other groups (*p* < 0.001), while differences among G2–G5 were not statistically significant. This pattern demonstrates a U-shaped relationship between eGFR stage and UA levels.

No statistically significant relationship was found between hypertension and prognosis (*p*-value = 0.197). Both hypertensive and non-hypertensive groups exhibited similar discharge rates and poor prognosis rates, suggesting that hypertension alone does not significantly affect the prognosis in this sample. Similar to hypertension, the presence of CAD did not show a significant effect on prognosis (*p*-value = 0.115). While 92.8% of patients without CAD were discharged, and 90.3% of those with CAD were discharged, the difference was not statistically significant.

Serum UA levels were significantly associated with prognosis. A higher proportion of patients with normal UA levels (93%) were discharged compared to those with low UA levels (87.8%). On the other hand, 12.2% of patients with low UA levels had a poor prognosis, compared to only 7% of those with normal UA levels. This relationship is statistically significant (*p*-value = 0.012), suggesting that low uric acid levels are linked to poor prognosis.

Table 2 shows the relationship between patient’s age, serum UA levels, and the presence of hypertension, CAD, and CKD with prognosis. It presents the results of a logistic regression analysis evaluating factors associated with mortality and prognosis based on serum UA levels. The analysis includes variables such as age, UA category, HT, CAD, and CKD.

Age was found to be a significant predictor of both mortality and poor prognosis. The positive coefficient (B = 0.034) indicates that with each additional year of age, the odds of poor prognosis and mortality increase. The Exp (B) value of 1.034 suggests that the likelihood of poor outcomes increases by approximately 3.4% for each year of age. This effect was consistently significant across all steps of the model (*p* < 0.05).

Serum UA levels were classified into categories, with low UA levels being associated with a significantly higher risk of poor prognosis and mortality. The B coefficient for UA category was 0.804, and the Exp (B) value was 2.236. This indicates that patients with low serum UA levels have more than twice the odds (about 123% increased risk) of having a poor prognosis compared to those with normal UA levels (*p* < 0.001). The association remained significant throughout the different model steps.

Hypertension was not found to be a significant predictor of poor prognosis in this cohort. The B value for hypertension was −0.284, with an Exp (B) of 0.753, indicating a 25% decreased risk of poor prognosis in hypertensive patients, although this finding was not statistically significant (*p* = 0.176).

The presence of CAD did not show a significant association with mortality or prognosis. The coefficient for CAD was very close to zero (B = 0.001), and the Exp (B) was 1.001, indicating negligible effect on the outcome. The *p*-value of 0.997 further suggests that CAD does not significantly contribute to the prognosis in this study.

CKD was associated with a higher risk of poor prognosis. The B coefficient for CKD was 0.535, and the Exp (B) value was 1.707, suggesting a 70.7% increased likelihood of poor prognosis in patients with CKD (*p* = 0.037). This effect remained significant after controlling for other variables in the model.

Our findings also demonstrate that the relationship between uric acid and renal function is nonlinear. Mean UA levels were lowest in patients with preserved renal function (G1), progressively increased in stages G2–G3, and then declined again in advanced CKD (stages G4–G5), forming a U-shaped curve. The decline in UA in advanced CKD may reflect malnutrition, reduced production, or impaired tubular handling of urate. Importantly, while hypouricemia was associated with poor prognosis in the overall cohort, subgroup analyses within each CKD stage did not reveal a significant prognostic effect of UA levels. This suggests that UA may act as a global prognostic biomarker across heterogeneous hospitalized populations, whereas its predictive role within specific CKD strata requires further validation.

In the regression model, age and serum UA levels were found to be the most significant predictors of poor prognosis, with serum UA levels (specifically low levels) being a particularly strong risk factor. The presence of CKD also contributed to worse outcomes, while hypertension and CAD did not show significant effects.

Our goal was to create a scoring system based on the contribution of variables to mortality risk and poor prognosis.

Multivariate logistic regression analysis identified the following as independent predictors of poor prognosis (ICU admission and/or death):

- Age: OR = 1.032 (95% CI: 1.021–1.043, *p* < 0.001).
- Low UA levels: OR = 2.285 (95% CI: 1.417–3.685, *p* = 0.001).
- CKD: OR = 1.571 (95% CI: 0.972–5.541, *p* = 0.065).

Neither hypertension nor coronary artery disease (CAD) were found to be statistically significant predictors of poor prognosis (*p* > 0.05), so these variables were excluded from the scoring system. Age significantly affects mortality, with the risk increasing each year so it was included as an important factor in the scoring system. Low UA levels also significantly increase mortality risk. Therefore, the scoring system was based on UA levels. The presence of CKD increases mortality risk and it was included as an additional risk factor in the scoring system.

Based on the logistic regression findings, we developed the Prognostic Uric Acid Score (PUAS) to stratify patients into risk categories. The scoring system assigns points based on key clinical variables significantly associated with poor prognosis. PUAS based on the variables is shown in the table below (Table 3).

**Figure 2:**
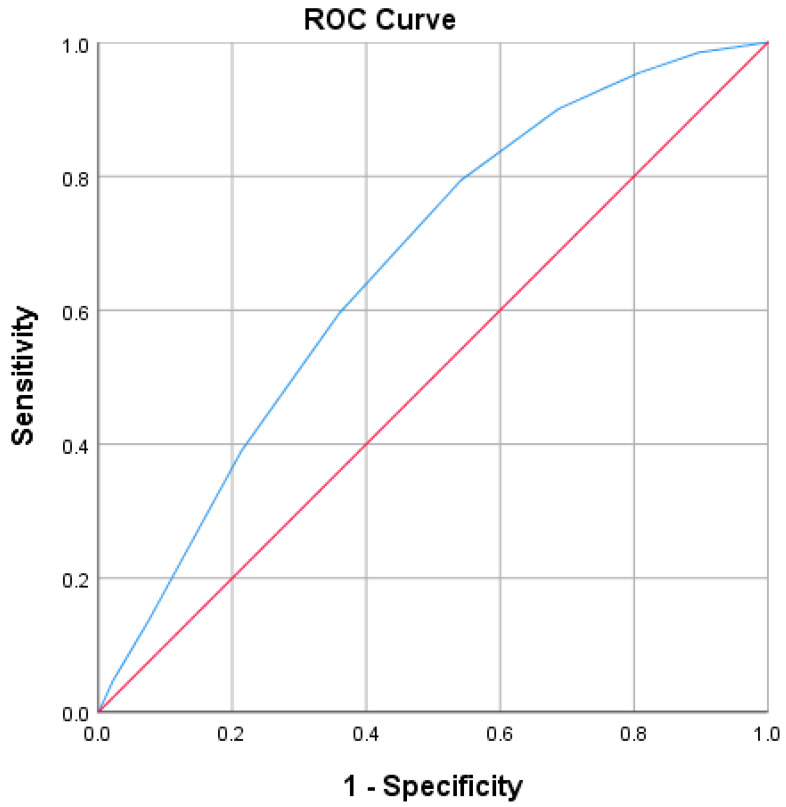
ROC Curve for PUAS. The blue line represents the ROC curve of PUAS, demonstrating the trade-off between sensitivity (true positive rate) and 1–specificity (false positive rate) across various thresholds. The red diagonal line represents the reference line of no discrimination (AUC = 0.5), corresponding to a random classifier. The area under the curve (AUC = 0.659, 95% CI 0.614–0.703, *p* < 0.001) indicates that PUAS has a modest but significant predictive value for poor prognosis.

The PUAS was validated using ROC curve analysis demonstrating an area under the curve (AUC) of 0.659 (0.614–0.703, *p* < 0.001), indicating a moderate predictive ability (Figure 2), (Table 4).

Based on the data and ROC curve analysis, the optimal cutoff value for predicting poor prognosis and high mortality risk was determined to be PUAS ≥ 3.5, using the Youden Index (J = 0.253). This threshold provides a balance between sensitivity (79.4%) and specificity (45.9%), offering a reasonable compromise between correctly identifying high-risk patients and maintaining an acceptable level of false positives. Although specificity is lower at this cutoff, the high sensitivity ensures that a significant portion of patients at risk for poor outcomes are identified, making the PUAS ≥ 3.5 cutoff particularly useful for screening purposes. This value prioritizes the detection of patients who may benefit from closer monitoring or early intervention. In contrast, a higher cutoff (e.g., PUAS ≥ 4.5) would increase specificity but at the cost of significantly reduced sensitivity (59.5%), potentially missing many patients at risk for poor prognosis. Therefore, PUAS ≥ 3.5 is recommended as the most appropriate threshold for stratifying patients based on their risk of adverse outcomes, offering a reliable prognostic tool for clinical decision-making.

## 4. Discussion

While hyperuricemia has been extensively studied in relation to cardiovascular and renal diseases, research on hypouricemia and its clinical implications remains limited. Although most studies define hypouricemia as a serum UA level < 2.5 mg/dL, there is no universally accepted threshold, and alternative cutoffs have been suggested based on population-specific characteristics.

UA has been recognized as a key factor in immune system regulation and tumor suppression, potentially due to its role as an endogenous antioxidant and its interaction with inflammatory pathways [12]. Hypouricemia has been reported in patients with malignancies, possibly as a result of increased oxidative stress, tumor-related metabolic alterations, or chemotherapy-induced effects [13,14]. Additionally, UA has a protective role in endothelial function, and its depletion has been linked to increased oxidative damage, vascular dysfunction, and a heightened risk of hypertension, diabetes mellitus, and chronic kidney disease [15].These findings suggest that serum UA levels may serve as a readily available and cost-effective biomarker for mortality risk stratification, particularly in hospitalized patients. Our study further revealed that age, CKD, and low UA levels were independent predictors of poor prognosis, reinforcing the role of UA in clinical decision-making. The presence of CKD was associated with a higher risk of poor prognosis.

Previous research has highlighted the prognostic relevance of serum UA levels across different populations. Two studies have demonstrated a U-shaped relationship between uric acid levels and mortality [16,17]. Another study conducted in Japan showed that optimal serum UA levels were associated with the lowest risk of cardiometabolic diseases [18]. In a 2021 study from the United States men with low serum UA levels had a 33% higher risk of all-cause mortality, whereas no significant relationship was observed in women [19]. Our findings support these observations by demonstrating a significant, gender-independent association between hypouricemia and increased all-cause and cardiovascular mortality. Furthermore, our study showed that both men and women with low UA levels had a significantly higher risk of mortality and poor prognosis, suggesting that discrepancies in previous reports may arise from differences in study populations, baseline characteristics, or gender-specific cutoff values.

Collectively, these studies support the notion that both low and high UA levels can influence clinical outcomes, emphasizing the importance of maintaining UA within an optimal range. Building upon these findings, our study focused specifically on hospitalized patients—a population less frequently addressed in prior research—and further confirmed that low UA levels are independently associated with poor prognosis and mortality.

These observations are supported by recent studies highlighting the dual role of uric acid in renal physiology and pathology and the potential clinical implications of modulating its levels. Hassan et al. reported that uric acid–lowering therapy is associated with the incidence of chronic kidney disease, highlighting the potential consequences of modifying serum uric acid levels in clinical practice [20]. Complementing these findings, a systematic review and meta-analysis demonstrating that reductions in uric acid levels can influence the progression of chronic kidney disease, underlining the importance of careful therapeutic strategies [21]. Additionally, a comprehensive overview of the interplay between uric acid and renal function, emphasize its complex pathophysiological mechanisms and potential as both a biomarker and therapeutic target [22]. Collectively, these studies reinforce the clinical significance of uric acid monitoring and management, supporting its consideration in risk stratification and prognostic assessment in patients with or at risk for kidney disease.

To facilitate clinical application, we developed the Prognostic Uric Acid Score (PUAS), a simple yet effective risk stratification tool incorporating age, CKD, and serum UA levels. With an optimal cutoff of ≥3.5, the PUAS provides a practical tool for risk stratification in hospitalized patients. This threshold prioritizes sensitivity (79.4%) to ensure that most high-risk patients are identified, facilitating closer monitoring or early intervention. While higher thresholds improve specificity, they reduce sensitivity and may overlook vulnerable patients. Integration of PUAS ≥ 3.5 into routine assessment may help clinicians identify patients at increased risk for adverse outcomes, pending further prospective validation.

This study has several limitations. Its retrospective, single-center design may limit generalizability and precludes causal inference. Although we adjusted for multiple confounders, residual confounding from unmeasured factors—such as diet, hydration status, physical activity, or genetic predisposition—cannot be excluded. Serum uric acid was assessed at a single time point, potentially overlooking temporal fluctuations. In addition, data on the use of SGLT2 inhibitors were not systematically available, preventing evaluation of their potential impact on uric acid levels. Since these medications can lower uric acid via the URAT9 transporter, they may have influenced our findings. Moreover, our dataset lacked detailed information on acute kidney injury (KDIGO stages I–III). As AKI can affect both uric acid levels and patient outcomes, this aspect should be considered in future studies.

Given the growing body of evidence linking hypouricemia to increased all-cause and cardiovascular mortality, well-designed prospective clinical trials are needed to further explore the role of UA in disease progression and to assess the potential benefits of therapeutic interventions aimed at optimizing serum UA levels.

## 5. Conclusions

Our study provides robust evidence that low serum uric acid (UA) levels are an independent predictor of poor prognosis and increased mortality in hospitalized patients. These findings reinforce the significant association between hypouricemia and adverse clinical outcomes, particularly in critically ill patients, highlighting the potential of serum UA as a simple and accessible prognostic biomarker in clinical practice.

Regular monitoring of serum UA levels, particularly in patients with chronic kidney disease and those at risk of cardiovascular complications, may facilitate early identification of high-risk individuals and enable proactive management strategies to mitigate adverse outcomes. Furthermore, our study underscores the need to integrate serum UA testing into routine clinical workflows, particularly for high-risk populations. The Prognostic Uric Acid Score (PUAS) we developed may serve as a valuable tool in guiding risk stratification and optimizing clinical decision-making.

Given the strong association between hypouricemia and poor prognosis, further research is needed to elucidate the underlying pathophysiological mechanisms, explore potential therapeutic targets, and determine whether interventions aimed at modifying serum UA levels could improve clinical outcomes. Well-designed prospective studies are crucial to validate our findings and assess whether UA modulation strategies could contribute to improved survival and long-term health outcomes.

Our findings provide practical recommendations for clinical practice. Low serum uric acid (UA) identifies hospitalized patients at higher risk of mortality and poor prognosis, particularly when combined with advanced age or chronic kidney disease (CKD). The Prognostic Uric Acid Score (PUAS), with a cutoff of ≥3.5, offers an effective tool for risk stratification, enabling the early recognition of high-risk individuals who may benefit from closer monitoring and timely interventions. Routine measurement of UA can thus serve as a simple, cost-effective biomarker to guide personalized patient management. Clinicians should consider hypouricemia as a meaningful prognostic indicator in hospitalized populations, while future prospective studies will be essential to validate and refine its role in clinical decision-making.

## Figures and Tables

**Table 1:** Patient Distribution Based on Key Clinical Variables and Prognostic Categories.

		Prognosis		
Variable	Category	Discharged *n* (%)	Poor Prognosis *n* (%)	*p*
**Age**		57 (17–96)	65 (18–91)	<0.001
**Chronic Kidney Disease(CKD)**	Absent	1372 (93%)	105 (7%)	0.015
	Present	176 (88%)	24 (12%)	
**Hypertension**	Absent	941 (93%)	71(7%)	0.197
	Present	606 (91.3%)	58 (8.7%)	
**Coronary Artery Disease (CAD)**	Absent	1241 (92.8%)	96 (7.2%)	0.115
	Present	306 (90.3%)	33 (9.7%)	
**Uric acid**	Normal (Male 3.4–7, Female 2.4–5.7)	1375 (93%)	105 (7%)	0.012
	Low (Male < 3.4, Female < 2.4)	173 (87.8%)	24 (12.2%)	

**Table 2:** Factors Associated with Mortality and Poor Prognosis Based on Serum Uric Acid Levels.

Variable	B	S.E.	Wald	df	Sig.	Exp (B)	95% CI for Exp (B)
**Step1**							
Age	0.034	0.006	32.968	1	0.000	1.034	1.023–1.046
Uric Acid Category	0.804	0.244	10.831	1	0.001	2.236	1.385–3.610
Hypertension	−0.284	0.210	1.828	1	0.176	0.753	0.499–1.136
Coronary Artery Disease	0.001	0.229	0.000	1	0.997	1.001	0.639–1.567
Chronic Kidney Disease	0.535	0.256	4.362	1	0.037	1.707	1.003–2.280
Constant	−4.068	0.412	97.559	1	0.000	0.017	
**Step 2**							
Age	0.034	0.006	33.666	1	0.000	1.034	1.023–1.046
Uric Acid Category	0.804	0.244	10.831	1	0.001	2.236	1.385–3.610
Hypertension	−0.283	0.205	1.918	1	0.166	0.753	0.504–1.125
Chronic Kidney Disease	0.535	0.253	4.463	1	0.035	1.707	1.039–2.804
Constant	−4.068	0.401	103.164	1	0.000	0.017	
**Step 3**							
Age	0.031	0.006	31.733	1	0.000	1.032	1.021–1.043
Uric Acid Category	0.826	0.244	11.478	1	0.001	2.285	1.417–3.685
Chronic Kidney Disease	0.452	0.245	3400	1	0.065	1.571	0.972–5.541
Constant	−3.909	0.384	103.704	1	0.000	0.020	

**Table 3:** Prognostic Uric Acid Score (PUAS).

Variable	Criterion	Score
Age	Every 10 years	+1
Uric Acid Category	Normal	0
	Low	+1
Chronic Kidney Disease (CKD)	Present	+2

**Table 4:** Area Under the Curve.

Area	Std. Error	Asymptotic Sig.	Lower Bound	Upper Bound
0.659	0.023	0.000	0.614	0.703

## Data Availability

The data presented in this study are available on request from the corresponding author. The data are not publicly available due to ethical restrictions.
